# An Assessment Model for Agricultural Databases: The Arthropod Pesticide Resistance Database as a Case Study

**DOI:** 10.3390/insects15100747

**Published:** 2024-09-27

**Authors:** Jane Payumo, Julia Bello-Bravo, Vineeth Chennuru, Solo Arman Mercene, Chaeyeon Yim, Lee Duynslager, Bhanu Kanamarlapudi, Omar Posos-Parra, Sky Payumo, David Mota-Sanchez

**Affiliations:** 1 Research Evaluation and Data Analytics, MSU AgBioResearch, Michigan State University, East Lansing, MI 48824, USA; 2 Department of Agricultural Sciences Education and Communication, Purdue University, Lafayette, IN 47907, USA; mbellobr@purdue.edu; 3 Department of Entomology, Michigan State University, East Lansing, MI 48824, USA

**Keywords:** impact assessment, global metrics, agricultural database, pesticide resistance, qualitative survey, quantitative analysis

## Abstract

**Simple Summary:**

This paper presents a multi-method approach to assess the utility and impact of agricultural databases, with a focus on the Arthropod Pesticide Resistance Database. It offers practical guidelines for developers, users, evaluators, and funders to assess such databases, highlighting key metrics for validating their impact. This paper introduces an index-based method that evaluates the database’s influence across several dimensions, including data usage, accessibility, knowledge generation, innovation, policy development, and collaboration. The approach serves as a reference model for assessing the impact of other agricultural databases.

**Abstract:**

This paper presents a multi-method approach for evaluating the utility and impact of agricultural databases in the context of the rapidly expanding digital economy. Focusing on the Arthropod Pesticide Resistance Database, one of the most comprehensive global resources on arthropod pesticide resistance, we offer a framework for assessing the effectiveness of agricultural databases. Our approach provides practical guidance for developers, users, evaluators, and funders on how to measure the impact of these digital tools, using relevant metrics and data to validate their contributions. Additionally, we introduce an index-based method that evaluates impact across multiple dimensions, including data usage, accessibility, inclusivity, knowledge generation, innovation, research and policy development, and collaboration. The detailed methodology serves as both a reference and a model for evaluating the impact of other agricultural databases, ensuring they effectively support decision-making and foster innovation in the agricultural sector.

## 1. Introduction

The digital revolution has dramatically increased the availability and sharing of agricultural data through structured databases. These databases, containing information on farming, crops, livestock, climate, and soil, have become essential for both public and private organizations involved in agriculture. They provide data on crops, weather, soil conditions, and market trends, aiding decision making and driving productivity, sustainability, and profitability [1]. Accessible databases also support precision agriculture, research, compliance, and market intelligence, contributing to modern agricultural practices [2,3,4]. 

An example is the Arthropod Pesticide Resistance Database (APRD), which tracks global cases of pesticide resistance and offers critical insights for mitigating its impacts on agriculture, public health, and the environment. Since 1914, APRD has recorded resistance data, covering 632 arthropod species resistant to 364 compounds, with 18,871 cases documented [5]. Pesticide resistance, especially in insects and arthropods, is a growing problem that led to the creation of APRD. Resistance occurs when pest populations develop genetic tolerance to pesticides due to repeated exposure [6], reducing pesticide effectiveness and increasing agricultural costs and losses [7]. Despite integrated pest management (IPM) efforts, resistance continues to emerge. Climate change and invasive species are expected to accelerate resistance further, threatening food security and public health [8,9,10,11]. The economic cost of resistance reaches billions, with additional impacts on pest management for crops, livestock, and human health [12,13]. By offering extensive data and analysis, APRD enhances understanding of the scale, distribution, and impacts of pesticide resistance, helping to accelerate efforts to manage this growing problem. 

Despite the importance of agricultural databases, structured research evaluating their broader impacts on stakeholders remains limited. Existing studies mainly focus on database design and technical features, for example [14,15,16,17,18,19], with little attention to factors like user engagement and stakeholder benefits. Beyond their primary role as data sources [20], the broader impacts and uses of agricultural databases remain underexplored. As the field of agricultural data management evolves, there is a growing need for data providers to go beyond merely making databases available. The effective use of these tools requires addressing factors such as accessibility and data quality [21], long-term commitment and sustainability [20,22], and openness and transparency [23]. Other factors include interoperability [24], privacy and security [25], ethical considerations [26], capacity building, and community engagement [27]. 

This paper addresses the research gap by using APRD as a case study to propose a framework for assessing the impact of agricultural databases. The framework evaluates dimensions, such as data usage, accessibility, knowledge dissemination, and collaboration, aiming to guide developers, users, and funders in measuring the success of these digital tools. Ultimately, this approach seeks to advance agricultural innovation and ensure that databases effectively meet the diverse needs of the sector’s stakeholders.

### 1.1. Agricultural Databases and IPM

An agricultural database is an organized collection of data stored and accessed electronically. It stores and manages structured and unstructured data on a subject/topic, individual, or organization. It offers rapid, precise, and integrated access to qualitative and quantitative information. Data from agricultural databases are available in digital or online formats and data types to address specific data goals and user needs. International organizations like the Food and Agricultural Organization [28] and the Consultative Group on International Agricultural Research [29] play significant roles in collecting, managing, and sharing agricultural data and statistics globally. National agricultural agencies in different countries also offer databases tailored to local agricultural needs. Universities and academic institutions also provide and manage agricultural databases for researchers, extensionists, students, policymakers, and the public. 

Common types of agricultural databases include those that relate to crops, livestock, soil, weather and climate, market and trade, genomic and genetic information, and farm management. Databases are also part of IPM efforts. There is now a plethora of databases for plant pests and diseases and database systems that handle small or large amounts of data tailored to support IPM-related projects [30] and the Pesticide Properties Database [31]. Six extensive and accessible online databases include the National IPM Database, the U.S. Environmental Protection Agency’s Registered Pesticides, the National Pesticide Information Center, the Pesticide Risk Tool, the University of California IPM, and the California Pesticide Information Portal [32]. These databases support implementing IPM strategies, including pest monitoring and surveillance, chemical identity, decision support, pest identification, chemical application management, and pest control. By consolidating and disseminating information on resistance mechanisms, geographic distribution, and control strategies, these databases empower stakeholders to make informed decisions, develop IPM, and combat the challenges of agricultural pests and diseases.

### 1.2. Impact Assessment of Agricultural Databases

Agricultural databases also serve as the foundation for rigorous and evidence-based impact evaluations—a systematic approach used to examine the utility, relevance, effectiveness, efficiency, and impact of an intervention, program, policy, or project concerning their stated objectives and wider aims [33]. They provide data and information that help ensure that assessments offer high-quality, well-organized insights and informed decisions on program effectiveness and broader impacts and benefits. 

Databases are essential in addressing the digital divide, which disproportionately impacts low-income groups, women, the elderly, and those with disabilities [34]. With over 80 percent of the population in the least developed countries still offline [35], the digital divide worsens inequalities across socioeconomic, gender, and geographic lines. Research advocates for more robust policies to promote digital inclusion, particularly for marginalized groups like women in agriculture, who benefit from access to digital tools and data [36]. Initiatives like Scientific Animations Without Borders (SAWBO), which provide educational materials in local languages, help empower women [37,38]. Despite progress in female representation in science and engineering [39], further efforts are needed to ensure gender-inclusive digital participation for broader development outcomes.

Beyond the digital divide and gender dimension topics, data from agricultural databases are paramount. Yet, impact studies on these databases per se are less prominent or less frequently reported. This can be due to several factors, including non-uniformity of the impact of agricultural databases: popular vs. smaller, less known databases, domain-specific research interests, regional differences in data preference, methodological challenges (especially on data quality and causality determination), impact as not being direct or immediate, and the need for an interdisciplinary approach to assess the impact of these databases comprehensively [20,40,41]. The level of analysis in the available literature also varies depending on the specific database, its scope, and its importance in agriculture. As the role of data in agriculture continues to grow, leading to an overwhelming pace of data and investments in setting up databases, we need to give more attention to assessing the added value and overall impact of agricultural databases. This paper is a response to this call. 

Assessing the impact of an agricultural database can encompass a wide range of considerations and perspectives. For this work, two theories and models provide valuable frameworks for evaluating the effects of APRD as an agricultural database. Based on the Information-seeking and Use Theory, the information-seeking behaviors of individual users helped understand the impact of farming databases as a source of information [42]. This framework explores how individuals search for, access, evaluate, and utilize information to meet their needs or accomplish their goals. In the context of agricultural databases, this theory provided us valuable insights into how APRD users and stakeholders engage with this resource to obtain relevant information and make informed decisions. Secondly, the Socio-technical Systems Theory, which explores how technology impacts and is influenced by social structures, helped us enhance our understanding of integrating agricultural databases into agricultural systems and practices [43]. Beyond social structures, this theory also suggests that isolation does not create technologies, but social factors, such as behaviors, cultural norms, economic interests, and power dynamics. Guided by these theories, we focused on multiple dimensions and areas (Table 1) to assess the impact of agricultural databases using APRD as a case study. As needed, this study used proxies and indicators to answer questions that are challenging to measure directly. 

### 1.3. Case Study: APRD

Managed at Michigan State University (MSU), APRD is a web-based agricultural database focused on pesticide resistance information. This database, https://www.pesticideresistance.org/ (accessed on 23 November 2023), was first developed in 2006 and was funded by the U.S. Department of Agriculture, Michigan Department of Agriculture, and MSU internal grants. This is a database of reports of pesticide resistance cases (including the time and place of discovery) collected from scientific literature, field studies, and monitoring programs from 1914 to the present. This online database also details species with documented resistance to unique pesticide formulations, mixtures, or compounds in one or more locations worldwide [7]. The database contains submissions of new resistance cases from experts worldwide. An editorial panel of case editors with multiple years of research experience and knowledge of pesticide resistance reviews these submissions for inclusion and reporting in the APRD. 

**Figure 1:**
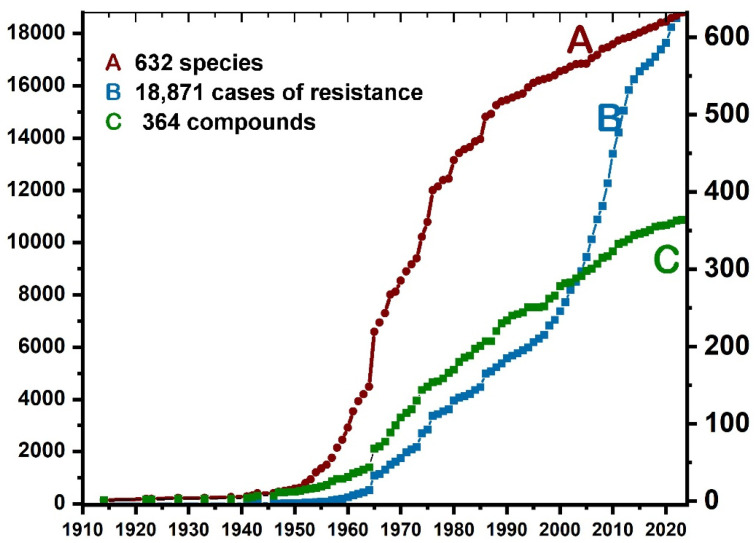
Evolution of pesticide resistance from 1914 to 2024. Species, compounds, and cases of resistance [5].

The APRD publishes up-to-date and dynamic summary tables with instantly updated information as new submissions are reviewed and published in the database. Multiple groups funded the APRD, including the U.S. Department of Agriculture’s Cooperative State Research, Education, and Extension Service Pest Management Alternatives Program, the Insecticide Resistance Action Committee (IRAC), and the Generating Research and Extension to Meet Economic and Environmental Needs (GREEEN) Project (#GR02-69). The database also obtained additional support from the Michigan Agricultural Experiment Station, Michigan State University Extension (MSUE), and the Michigan Department of Agriculture and Rural Development. The APRD now has more than 632 species and about 18,871 cases of pesticide resistance (Figure 1). 

Scientists and growers from the U.S. and worldwide can search a case of resistance in this database by year and type of resistance (field-evolved resistance or laboratory selected) and collect data and information by species, active ingredient, and country location. Specifically, the database offers searching based on three levels of hierarchy: order, family, and species. This information supported resistance monitoring analyses [50] and reporting for pesticide registration, pesticide reregistration processes, and recommendations in resistance management [51]. This database also allows for the identification of patterns and trends in resistance development, aiding in developing targeted and effective pest control measures. A review by Dialynas et al., 2009 [19], indicated that APRD’s reports of instances of resistance are useful as general indicators of pesticide resistance. According to this review, however, it lacks geographic accuracy, combined with the lack of a map interface, making it less suitable as a tool either by itself or with a modern, IT-based decision support system. However, geographic accuracy is determined by the information provided in published articles. In addition, farmers often dislike descriptions of their farms as sources of resistant insect pests. 

Regularly reviewing and evaluating agricultural databases like APRD is crucial for ensuring their utility, accuracy, and relevance. These evaluations help identify data gaps, emerging needs, and opportunities for technological improvements, enhancing efficiency and accuracy. They also provide insights into user interactions, challenges, and preferences, guiding user interface and experience improvements. Demonstrating the positive impact of these databases supports continued funding and backing from stakeholders, donors, and government agencies.

## 2. Materials and Methods

We employed a multi-method approach, combining qualitative and quantitative approaches, to review and evaluate APRD and its impact from November 2023 to February 2024. 

Web analytics tools, such as Google Analytics and user-engagement data, helped understand the database usage for 2018–2022. User-engagement data collected included website traffic, unique visitors, and demographic information, including age, gender, geographical location of users, and device type. Other assessments include the accessibility of APRD across various devices, search functionality (interface), user support, user-friendliness, and performance in retrieving relevant information from the pesticide resistance database. 

A four-question feedback form was designed in Microsoft Forms and integrated into the APRD website during the study period. A discreet banner invited users to participate, highlighting confidentiality and the purpose of enhancing user experience and improving services. The feedback form was accessible via a direct link on the website, and anonymous feedback was collected without requesting personal information. Website visitors were asked the following:

1. Overall, how would you rate your experience using the arthropod resistance database?
2. Please specify the primary purpose for which you used the arthropod resistance database.
3. Has the arthropod resistance database enhanced your research or work in any of the following ways? Check all that apply: (1) improved species identification, (2) enhanced understanding of arthropod distributions, (3) facilitated analysis of ecological interactions, (4) supported the assessment of arthropod resistance to insecticides, (5) informed pest management decisions, (6) Other: Please explain.
4. Is there any additional feedback or suggestions you would like to provide regarding the arthropod database?

Multiple publication databases served as data sources to collect scholarly literature related to APRD, including primary research, publications, and policy documents citing the use of APRD in various contexts. This information helped map the contribution of APRD to knowledge dissemination, innovation, research advancement, policy development collaboration, and networking. This query: (ALL (“pesticideresistance.com”)) OR (ALL (pesticide AND resistance AND database) informed the Python library to pull, cache, and extract data from the Scopus database, the largest abstract and citation database for academic research. The document types analyzed included articles, book/book chapters, conference/proceedings, reviews, editorials, notes, and short surveys. The Scopus IDs of each APRD developer (*n* = 5) helped collect all APRD-related publications by the developers. Relevant metadata collected on these publications includes the bibliographic and citation information, citation count, citation impact, abstract and keywords, and funding information. The number of authors, author affiliation address, disciplines, and topic/themes helped understand and visualize research collaboration, type of research collaboration (domestic or international), and themes/topics linked to the publications citing APRD. 

Altmetrics provided an additional understanding of the impact and reach of publications linked to APRD beyond the traditional citation metrics. Dimension (app.dimensions.ai; accessed 17 January 2024) provided the social and online impact of scholarly publications generated by APRD developers. To check on whether research that used APRD data is being used in policy development, Overton (https://www.overton.io/; accessed on 17 January 2024), the largest searchable index of policy documents, guidelines, think tank publications, and working papers, was also used in this study. Additionally, web scraping and reverse internet search for the APRD’s website 1 (“pesticideresistance.org”) helped identify and retrieve the top 100 websites that cited APRD. Finally, Python scripts to query Google Search to identify websites promoting and citing APRD helped automate the data extraction process and data organization. 

Tableau Prep (2023.3) helped us in the data cleaning and data preparation for bibliometric analysis, relational modeling, processing, descriptive statistics, and visual analytics in Tableau Desktop (ver. 2023.2.1). Additionally, VosViewer (ver. 1.6.20) helped visualize and understand the collaboration network (based on co-authorship) and the themes/topic clusters (based on co-occurrence) involved in the publications that cited APRD. The VosViewer software helps visualize similarities between articles, authors, or terms [52]. 

APRD is a global database that aims to impact an international audience. Calculating an impact index helped us understand whether APRD serves this purpose. This index, which equally weighs various factors, seeks to represent the overall global impact of APRD. It identifies which countries benefit most from APRD regarding database usage, publication output, citations, collaboration, and contributions from research to policy. The resulting index score for each country ranged from 0 (lowest impact) to 1 (highest impact). 

Significant efforts facilitated the collection and analysis of the various datasets to extract valuable insights into the reach, influence, and impact of APRD. Still, due to limitations (i.e., indexing in databases and search engines), these analyses for APRD are neither exhaustive nor comprehensive. It is hoped that the mixed-method approach, the data, and information supporting select impact metrics for agricultural databases and all insights generated from this assessment will serve as a reference for other groups wanting to track and analyze the impact of their databases using the same or a modified version of the approaches mentioned in this study. 

## 3. Results and Discussion

In this study, we meticulously examined the multidimensional effect of the APRD using mixed-method evaluation approaches. The results provide valuable insights into the implications of adopting this database and shed light on its broader influence and impact. The following discussion provides the key findings, their implications, and the database’s wider impact and reach. 

### 3.1. Database Usage, Accessibility, and Inclusivity

**Figure 2:**
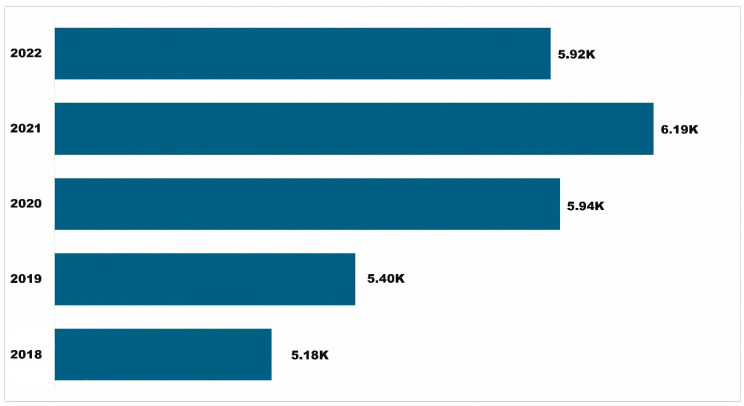
User growth chart for APRD, 2018–2022.

One of the notable outcomes of this assessment is finding an increasing number of diverse users and stakeholders of APRD over time (Figure 2). From 2018 to 2022, APRD had more than 28,600 users, growing at a compound annual growth rate (CAGR) of 4 percent annually. The year 2021 had the highest number of users, while 2020 (COVID-19 pandemic) had the highest year-over-year growth (7.65 percent). This notable achievement reflects APRD’s relevance and positive reception within its user community. 

**Figure 3:**
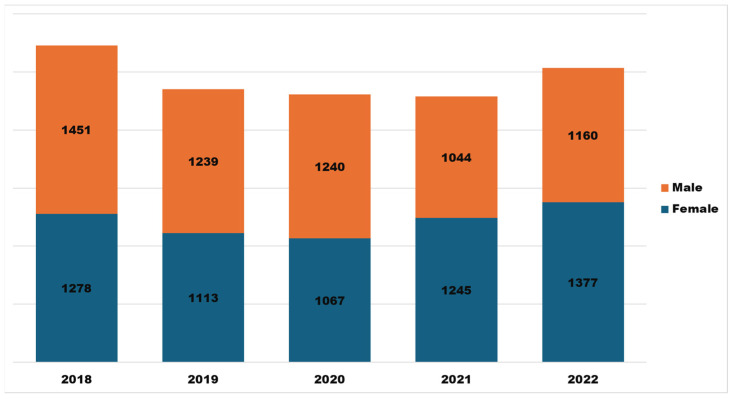
Gender distribution chart for APRD, 2018–2022.

More male users (*n* = 6134) accessed APRD than their female counterparts (*n* = 6080). However, the female user base of APRD has exhibited consistent growth over time, with a CAGR of 2 percent every year (Figure 3). This indicates an encouraging and steady rise in women’s interest and participation in accessing pest resistance data and information. 

**Figure 4:**
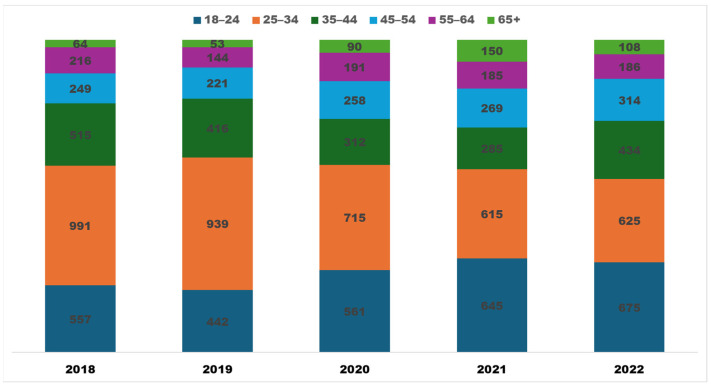
Age distribution profile of APRD users, 2018–2022.

APRD users represent all age groups (Figure 4), reflecting a diverse and inclusive audience engaged in accessing pest resistance data and information across different demographic segments. APRD, however, has witnessed growth in users only for these age groups: 65+, 44–54, and 18–24. This growing demand for APRD from these age groups and not for other groups has implications for user interface design, content delivery, and outreach strategies to cater to a broad demographic range. APRD has also experienced the highest surge in users aged 65+ (CAGR = 13.98 percent), suggesting a noteworthy trend of increased digital adoption among older individuals. This also indicates improved digital literacy and the growing recognition of the value of online resources for this age group. 

**Figure 5:**
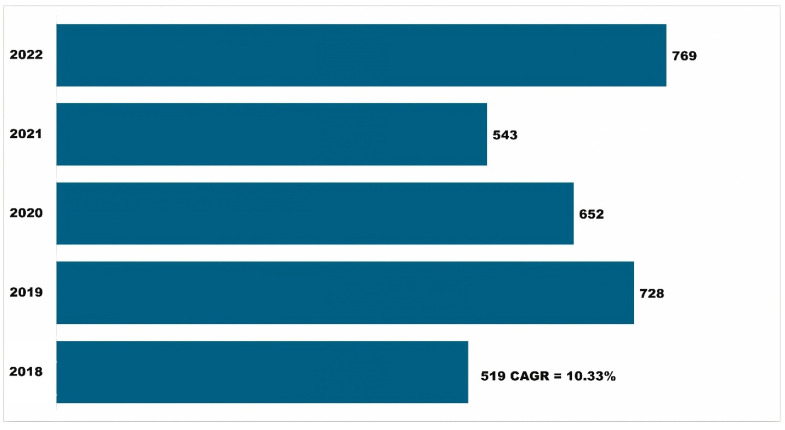
Growth in devices accessing APRD over time, 2018–2022.

The number of devices used to access APRD has also increased over time (CAGR = 10.33 percent). The highest number of devices recorded accessing APRD was in 2022 (Figure 5). The increasing number of devices accessing APRD over time again supports a growing and diverse user base, indicating a higher demand for pest resistance data and information across various platforms. This trend offers multiple implications, including increased accessibility, technology adoption, and user convenience. It also indicates a dynamic and evolving landscape, emphasizing the need for adaptability, user-focused design, and continuous improvement to meet the diverse needs of users in the agricultural community. 

### 3.2. Database Key Outcomes and Impacts

The qualitative and quantitative analyses provided valuable information on APRD’s key outcomes and impact. The initial results of the feedback questionnaire on APRD have been overwhelmingly positive, underscoring this resource’s significant impact and utility in the scientific community. 

### 3.3. Initial User Feedback Results

**Figure 6:**
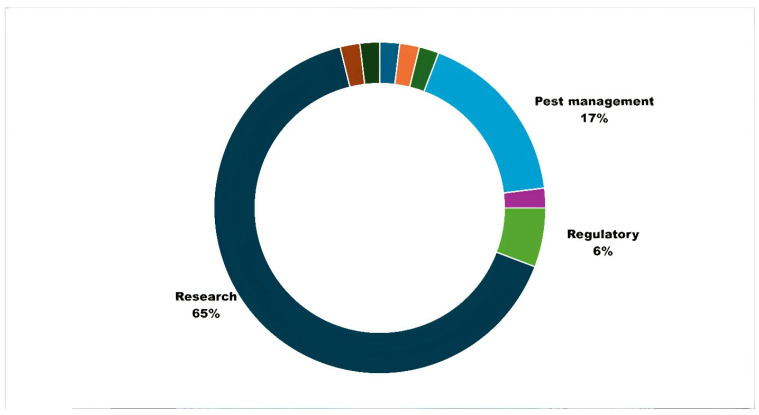
Primary reasons for accessing APRD.

**Figure 7:**
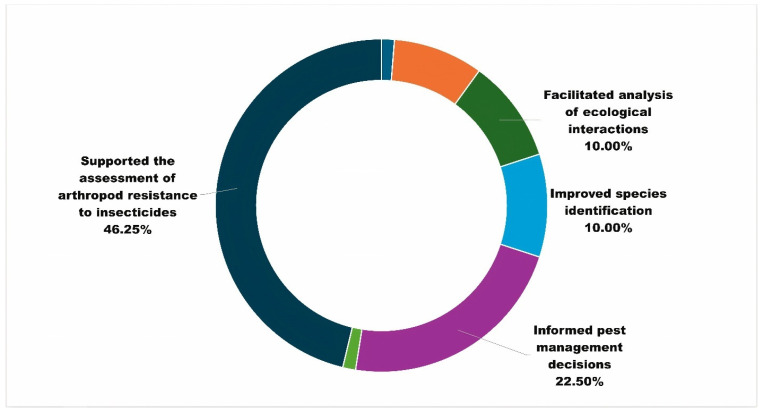
Contributions to research as identified by surveyed users (*n* = 52).

The results relating to initial user feedback (*n* = 52) validated the primary purposes of APRD as supporting research and pest management (Figure 6). Most surveyed users also indicated top reasons (Figure 7) for using the database, and these reasons included assessing arthropod resistance (46.25 percent), helping inform pest management decisions (22.50 percent), facilitating analysis of ecological interactions (10 percent), and improving species identification (10 percent). Researchers from various fields have praised the database for offering public access and comprehensive information on pesticide resistance. It facilitates their investigations into evolving resistance patterns among agricultural pests and management recommendations to commercial agricultural producers. These promising findings underscore the database’s role as a valuable tool in supporting and advancing research efforts and adding depth to the context supporting the numbers and trends observed in quantitative analyses (described next). As the APRD team continues to collect and analyze the user feedback data, we anticipate gaining further insights into other specific ways researchers leverage this resource to enhance their studies for sustainable pest control practices. Users provided suggestions to improve the user experience: sorting and exporting information, additional information on active ingredients linked to standardized chemical identity to allow for relevant search queries, comprehensive trend analysis of insecticide resistance data every year, and the addition of photographs.

### 3.4. Knowledge Generation and Dissemination

**Figure 8:**
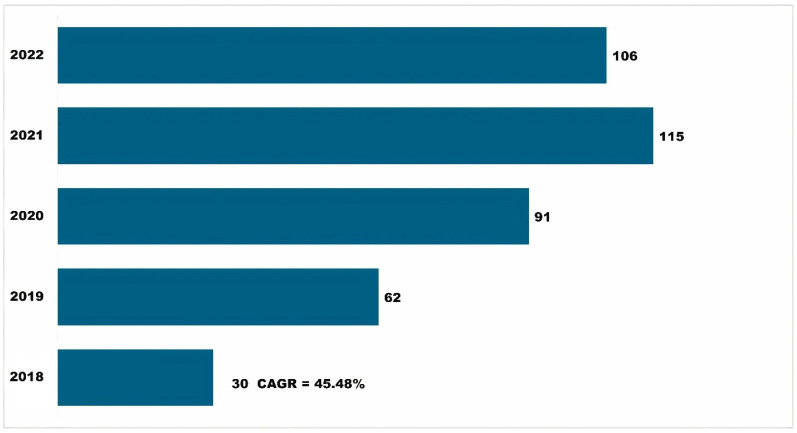
Growth in the number of publications citing APRD and contributing to knowledge generation and dissemination.

Over time, knowledge generation and dissemination involved developing and sharing information and insights using APRD to reach a broader audience. Research findings, best practices, and relevant APRD data are available in scholarly publications, gray literature, and online resources. A total of 399 scholarly publications (Figure 8) cited the use of APRD in their research and are available in scientific journals and academic articles. These scholarly publications have increased over time and grew at a CAGR of 43.48 percent. Other reports and various online resources also help promote the use of APRD for IPM and the knowledge and information from APRD. These knowledge dissemination efforts through scholarly publications, reports, and online resources reached the U.S. but also went to a diverse international audience, transcending geographical boundaries. 

**Figure 9:**
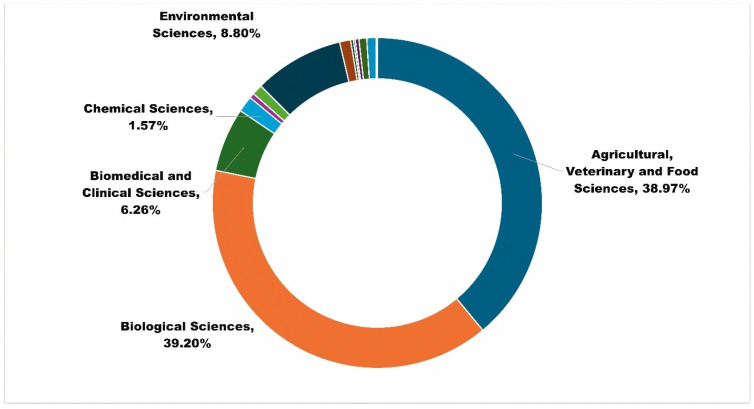
Research fields that find APRD as relevant.

The three APRD publications, Mota-Sanchez et al., 2008; Whalon, Mota-Sanchez, and Hollingworth, 2008; and Whalon, Mota-Sanchez, and Hollingworth, 2008, over time have received interest based on altmetrics data. From 2018 to 2022, these publications received 137 citations, with 13 percent received in the past two years, which is higher than expected. The citation impact of these publications is also 10.78 times higher than the global average (field-weighted citation impact equals 1.0). Experts working in agricultural, veterinary, and food sciences, which is APRD’s target audience, cited APRD-linked publications. Experts from other fields, such as the biological, chemical, environmental, biomedical, and clinical sciences (Figure 9), also found APRD publications relevant to their research. 

### 3.5. Innovation and Research Advancement

**Figure 10:**
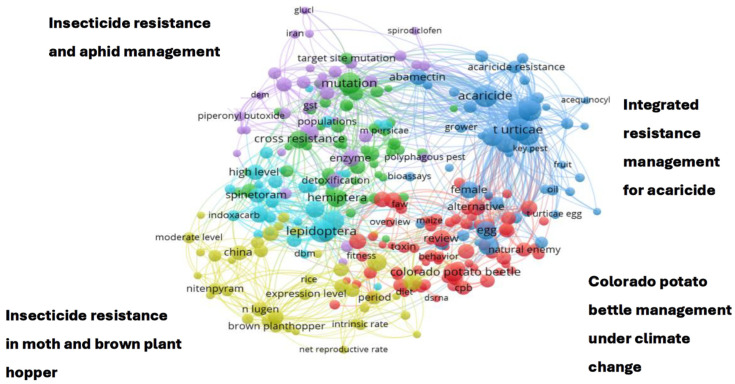
Key themes and research topics linked to APRD citations, 2018–2022.

**Figure 11:**
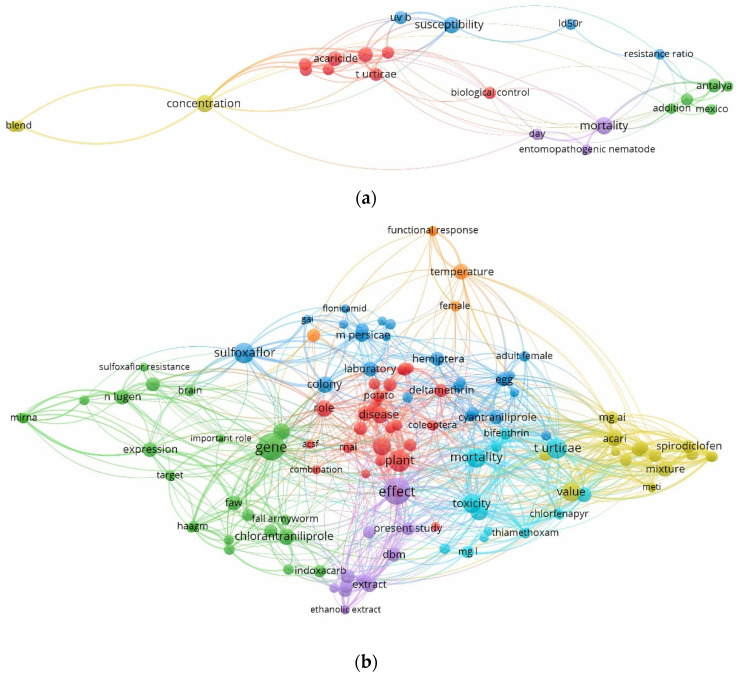
Comparison of key themes and research topics linked to APRD citations (**a**) 2018 versus (**b**) 2022.

This study used text analytics to investigate the research topics associated with scholarly outputs that cited the agricultural database APRD. Key themes and research topics observed for the 2018–2022 scholarly outputs that cited APRD include Colorado potato beetle (*Leptinotarsa decemlineata*) management under climate change, insecticide resistance and aphid management, integrated resistance management for acaricide, insecticide resistance in brown planthopper (*Nilaparvata lugens*) and moth (Figure 10). The analysis also revealed an increase in the number of key themes and research topics linked to citations of APRD, indicative of growing interest and utilization of this valuable resource from diverse experts within the scientific community. In 2017, experts cited APRD in research related to IPM concentration, management of red spider mites (*Tetranychus urticae*), tick and mites, and mosquito control. In 2022, the number of research topics has increased, and APRD was cited by research on the effects of broad-spectrum insecticide, fall armyworm (*Spodoptera frugiperda*), biopesticides, pesticide efficacy, and residual effects, resistance to Colorado potato beetles, insecticide and acaricide mixtures and effect of temperature on the functional response of predator–prey interactions. The increase in research topics underscores the role of APRD in shaping progress within the agricultural sciences and its contribution to understanding the dynamics of agricultural innovation and its use to advance agricultural research. Figure 11a provides a visualization of the research topics associated with citing APRD in 2017, while Figure 11b is the research topics related to citing APRD in 2022 (Figure 11b). The comparison highlights a dynamic and evolving research landscape in APRD, with a clear trend towards greater complexity and interconnectivity in research themes over time. In 2017, the network was more concentrated, with fewer clusters and less interconnectedness between them. Key themes were more centralized, focusing on a limited set of research topics within APRD. In 2022, there is a marked increase in the number of clusters, suggesting that new themes and issues have emerged. The network has expanded, with more connections between clusters, indicating that research in APRD has become more interdisciplinary and diverse. This growth may reflect broader participation in APRD and evolution in the areas of focus due to advancements in technology, changes in societal needs, or shifts in academic interest.

APRD served as a valuable resource for university inventors, researchers, and companies in the agricultural sector. Analysis of patent data revealed six patent applications filed in 2018–2022 and cited APRD. These patents relate to inhibiting insecticide resistance, pest control composition and applications, insecticide for moth control, and pogostone as an insecticide, repellant, and/or herbicide and were submitted by inventors from the US, Japan, and Brazil. 

### 3.6. Policy Development

APRD has helped inform agricultural policies and decision making. Policy documents by intergovernmental organizations such as the United Nations Environment Programme and the Intergovernmental Science-Policy Platform on Biodiversity and Ecosystem Services cited APRD. These policy documents relate to pesticide and pesticide resistance and soil and natural resource management. Specifically, they cited APRD on the increasing number of cases of resistance against species of insects and mites and that these cases are field-evolved (i.e., the result of pesticide use under field conditions rather than in the laboratory). The inclusion of APRD in these policy documents underscores its contribution to the information and knowledge base related to minimizing the environmental and health impacts of the use of pesticides and fertilizers. 

### 3.7. Collaboration and Networking

As the agricultural landscape becomes increasingly complex, research collaboration becomes essential for addressing global challenges affecting the agriculture sector. Publications that used and cited APRD involved research collaborations. The collaborative network comprised 18 clusters in 2018, then grew to 67 in 2022. However, a further look at the research collaborations at both time points reveals disconnected, individual research collaborative networks. The potential reasons behind this lack of connectivity and its broader implications are worth investigating and promoting the integration within this network and expanding it are worth exploring. 

### 3.8. Overall Impact

**Figure 12:**
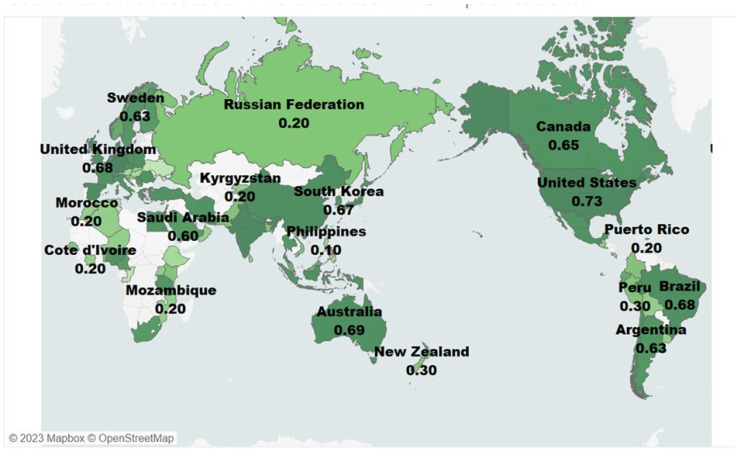
Global impact map: Countries benefiting from APRD use. The number next to each country represents its impact index score, calculated from the included metrics. A darker green indicates a higher score, ranging from 0 (lowest impact) to 1 (highest impact).

The study reveals patterns of APRD d usage across 87 countries, which helps validate the global reach of this database. Forty-one out of eighty-seven countries (47.12 percent) that cited APRD involved collaborative research. Citation analysis provides insights into the academic influence of APRD in 43 out of the 87 countries. On the other hand, no evidence shows the integration of database-driven research into policy-specific countries. The top 10 countries that used APRD the most include Germany, India, the United States, Israel, Belgium, the Czech Republic, Spain, China, Australia, Brazil, and the United Kingdom (Figure 12). All these countries have pesticide resistance cases in APRD, with the US, China, Australia, India, and Brazil having the highest cases. This result can inform future outreach and dissemination activities on APRD and its application and benefits to researchers in countries with increased pesticide resistance cases who use the database. 

## 4. Conclusions and Next Steps

The connection between agricultural databases, pest management, and agriculture is crucial for sustainable crop production, reduced environmental impact, and increased food security. Arthropod pesticide resistance databases such as APRD are invaluable tools in the realm of pest management in agriculture and promoting resilient agricultural systems. The importance of arthropod pesticide resistance databases lies in their ability to consolidate and disseminate valuable information, enhance collaboration, guide decision-making, and support sustainable pest management practices. APRD, a globally recognized resource, contributed to these objectives. APRD users have expressed appreciation for the database’s role in supporting research activities and its contribution to informed decision making in pest management strategies. However, databases such as APRD need to adapt to user preferences, ensure timely updates of relevant information (e.g., IRAC Mode of Action classification), and improve navigation and efficient retrieval of data so they continue to be effective and offer a user-friendly system. APRD’s implementation of design iterations over time and until recently proves that it is adaptable, user-focused, responsive, inclusive, and committed to continuous improvement of the database to meet the evolving needs and preferences of its user base. 

This paper provided a versatile toolkit for assessing the impact of agricultural databases, enabling developers, users, evaluators, funders, and other stakeholders to make informed decisions about developing and enhancing these digital resources for their user base. Using the APRD as a case study, we demonstrate the practical implementation of our evaluation methods, combining quantitative and qualitative approaches, providing a more holistic view and insights into the impact of this database. The use of website traffic tools such as Google Analytics helped in understanding the demographic information, including age, gender, and location of the users interacting with the database. The results helped establish the user base, engagement, and accessibility of the database as a digital tool and environment for all genders across age groups and device types. The use and integration of bibliometric, webometric, data mining, and qualitative techniques gave us an understanding of how APRD influenced knowledge creation, research advancement, innovation, collaboration, and policy development. Finally, introducing an index-based approach represents a significant contribution to the field. This approach evenly weighs the impact of agricultural databases across critical dimensions, such as data usage, accessibility, inclusivity, knowledge generation, dissemination, innovation, research, policy development, and collaboration, providing a nuanced understanding of their overall effectiveness. 

All of these are also valuable to APRD’s website owners and developers, who can shape future strategies for the database, further create a more inclusive, user-friendly experience, improve database features, optimize performance, and maximize its positive influence. These significantly facilitate increased interdisciplinary and transdisciplinary research collaborations and networking. These can be adapted and applied to other agricultural databases, fostering a standardized and systematic approach to impact assessment within the agricultural domain. 

## Figures and Tables

**Table 1:** Impact areas, their definitions, research questions, and indicators used in the study.

Dimension	Definition	Research Questions	Indicators	References
Database Usage, Accessibility, and Inclusivity	Users interact with APRD through querying and/or reporting new pesticide resistance. These users can access and retrieve the data from APRD by various groups, including women and marginalized communities.	How widely accessible is the database? What is the number of visits, downloads, or registered users? What user groups in terms of gender and age? And which device(s) were used to access the database? Which countries?	Database usage Search functionality Geographical access	[44]
Knowledge Generation and Dissemination	Sharing or distributing information contained in APRD to a wider audience for education and capacity-building	Are there any research publications or studies that directly cite the database as a resource in their research? Did the database facilitate the generation and dissemination of valuable knowledge and research findings to various stakeholders in the agricultural sector?	Publication and citation counts Social media shares and mentions	[45]
Collaboration and Networking	Collective knowledge, resources, and connections that APRD developers and users have established	Are there examples of collaborations or partnerships highlighting the use of the database?	Co-authored publications (domestic or international)	[46,47]
Innovation and Research Advancement	Contribution of APRD to agricultural innovation, research, and the development of technologies or practices within the field of pesticide or IPM research	Has the database facilitated new or emerging research themes related to IPM? Are there any indications that the database has influenced the development of new inventions and innovations for managing pesticide resistance?	Publication and citation counts Patent counts	[48]
Policy Development	Impact or effect that data and information gathered from APRD have on policies, development of policy, or the process of making decisions	Are there examples of instances where the database has informed policy decisions or regulatory actions related to pesticide use?	Policy document counts	[49]

## Data Availability

The original data presented in the study are openly available at https://doi.org/10.17632/jybbbr7m5x.1. The Python script to extract the top 100 websites that cited APRD is available at: https://github.com/vineethchennuru/Web-Scraping/blob/main/Reverse%20Internet%20search.ipynb (accessed on 6 February 2024).
